# Robotic versus laparoscopic distal gastrectomy in patients with gastric cancer: a propensity score-matched analysis

**DOI:** 10.1186/s12893-021-01212-4

**Published:** 2021-04-21

**Authors:** Taro Isobe, Naotaka Murakami, Taizan Minami, Yuya Tanaka, Hideaki Kaku, Yuki Umetani, Junya Kizaki, Keishiro Aoyagi, Fumihiko Fujita, Yoshito Akagi

**Affiliations:** Department of Surgery, Kurume University School of Medicine, 67 Asahi-machi, Kurume, Fukuoka 830-0011 Japan

**Keywords:** Robotic gastrectomy, Laparoscopic gastrectomy, GC, Short‐term outcomes

## Abstract

**Background:**

Robotic distal gastrectomy (RDG) has been increasingly used for the treatment of gastric cancer (GC). However, whether RDG has a clinical advantage over laparoscopic distal gastrectomy (LDG) is yet to be determined. Thus, this study aimed to assess the feasibility and safety of RDG for the treatment of GC as compared with LDG.

**Methods:**

In total, 157 patients were enrolled between February 2018 and August 2020 in this retrospective study. We then compared the surgical outcomes between RDG and LDG using propensity score-matching (PSM) analysis to reduce the confounding differences.

**Results:**

After PSM, a clinicopathologically well-balanced cohort of 100 patients (50 in each group) was analyzed. The operation time for the RDG group (350.1 ± 58.1 min) was determined to be significantly longer than that for the LDG group (257.5 ± 63.7 min; *P* < 0.0001). Of interest, there was a decreased incidence of pancreatic fistulas and severe complications after RDG as compared with LDG (*P* = 0.092 and *P* = 0.061, respectively). In addition, postoperative hospital stay was statistically slightly shorter in the RDG group as compared with the LDG group (12.0 ± 5.6 vs. 13.0 ± 12.3 days; *P* = 0.038).

**Conclusions:**

Our study confirmed that RDG is a feasible and safe procedure for GC in terms of short-term surgical outcomes. A surgical robot might reduce postoperative severe complications and length of hospital stay.

## Background

Gastric cancer (GC) has been identified as one of the most common causes of cancer-related mortality worldwide [1]. Radical gastrectomy with regional lymphadenectomy is still considered as the only curative treatment for GC [2, 3]. Since Kitano et al. first performed laparoscopically assisted gastrectomy for GC in 1991, minimally invasive surgery with laparoscopy has gradually been accepted as a common surgical procedure for GC patients [4]. Several reports have shown the advantages of laparoscopic distal gastrectomy (LDG) as compared with open gastrectomy, which include less invasiveness and pain, better cosmetic outcomes, rapid return of gastrointestinal function, and shorter hospital stay [5–7]. However, the current laparoscopic procedure has been determined to have some technical limitations, including two-dimensional visualization, straight rigid instruments, amplified physiologic tremor, and the uncomfortable position required for surgeons. The da Vinci surgical system (Intuitive Surgical, Inc., Sunnyvale, CA), which provides surgeons with high-resolution three-dimensional images, wristed instruments offering seven degrees of freedom, and tremor filtration technology, has been identified to be capable of overcoming the lack of perspectives and maneuverability restrictions in laparoscopic surgery. In 2003, Hashizume and Sugimachi were the first to use robotic gastrectomy (RG) for the treatment of GC [8]. In April 2018, RG was approved for national medical insurance coverage in Japan; since then, the number of RG procedures performed in this country has seen a significant increase [9]. Several studies have compared the safety and feasibility of robotic distal gastrectomy (RDG) and LDG [2, 10, 11]. However, most of these studies did not take all the background data into consideration. Therefore, in terms of safety and feasibility, whether RG should be recommended as the standard surgical treatment for GC is still controversial. Thus, this study aimed to assess the short-term surgical outcomes between RDG and LDG using propensity score-matching (PSM) analysis to adjust for patient background data as far as possible.

## Methods

### Patients

This present study was performed in accordance with the principles of the Declaration of Helsinki. This study was approved by the ethical committee of Kurume University (approval No. 19201). All patients provided a written informed consent for their operations. We retrospectively collected data from a prospective database of Kurume University Hospital. We used the following as inclusion criteria for the study: all patients diagnosed with GC via pathological biopsy and those with clinical T1-4N0-3M0 GC based on the Japanese classification of gastric carcinoma (JCGC) [12]. We then excluded the following patients: those with indications for endoscopic submucosal dissection, those with large (≥ 8 cm) macroscopic type 3 cancer or type 4 cancer and bulky lymph node (LN) metastasis, those requiring splenectomy, those with distant metastasis and remnant stomach cancer, and those who had prior chemotherapy or radiotherapy for any malignancy or prior upper abdominal surgery or intestinal resection other than appendectomy or cholecystectomy. Tumors were classified according to the JCGC, and terminology was also used in accordance with the JCGC.

Between February 2018 and August 2020, we were able to perform RDG in total 69 patients and LDG in 88 patients with GC at Kurume University Hospital. Clinicopathological characteristics [such as age, gender, body mass index (BMI), tumor status, American Society of Anesthesiologists performance status (ASA-PS) classification, and comorbidity] and short-term outcomes [including operative time, blood loss, conversion to another method, extent of resection, reconstruction, extent of LN dissection, postoperative morbidity and mortality, postoperative hospital stay, maximum plasma C-reactive protein (CRP) levels, and drain amylase (D.AMY) concentration in the drainage fluid at postoperative day 3] were also determined and compared between the two groups. Postoperative complications were considered for those with Clavien–Dindo classification (CDC) grade II or greater that occurred within 30 days after gastrectomy [13]. If a patient had multiple complications, we utilized the complication with the highest grade for the analysis.

### Surgical procedures

All gastrectomy procedures were performed according to the standard of radical gastrectomy based on the Japanese GC treatment guidelines [14]. The procedures employed during RDG are not different from those of LDG except for the use of articulating robotic instruments. During surgery, patients were placed in a supine and reverse Trendelenburg position, with legs elevated approximately 15°–20°. Both surgical procedures used a total of five trocars. RDG was performed using the da Vinci surgical system Xi with four articulating arms: one central arm for a 30° rigid dual-channel endoscope and the remaining three for a Cadiere forceps, fenestrated bipolar forceps, and bipolar Maryland forceps or Vessel Sealer system. One assistant port was placed at the right umbilical level. When Billroth-I reconstruction was technically possible, we used a Delta-shaped anastomosis. When Billroth-I reconstruction could not be used, we applied instead Billroth-II or Roux-en-Y reconstruction based on the preference of the operating surgeon. A single abdominal drainage tube was inserted into the left subphrenic cavity after reconstruction.

### PSM analysis

We performed PSM analysis to remove the confounding factors and overcome possible patient selection bias. For all patients, the propensity score was calculated using a logistic regression model based on the following variables: age, gender, BMI, ASA-PS, history of abdominal operation, comorbidity, tumor size, histology, pT stage, pN stage, and pTNM stage. A 1:1 nearest-neighbor matching algorithm with an optimal caliper width of 0.20 without replacement was used in matching the propensity score. After PSM, the balance of variables between the two groups was assessed by calculating the standardized mean difference (SMD). An SMD < 0.10 of the absolute value was considered to be a sufficient balance.

### Statistical analysis

All statistical analyses were performed using the JMP software, version 14 (SAS Institute Inc., Cary, NC, USA). Categorical variables were compared using the Pearson’s χ^2^ test or Fisher’s exact test, whereas normally distributed continuous variables were expressed as mean ± standard deviation and analyzed using Student’s *t*-test. Nonparametric continuous values were expressed as median (interquartile range) and were compared using the Mann–Whitney *U* test. A *p*-value < 0.05 was considered to be statistically significant.

## Results

### Clinicopathological characteristics

From February 2018 to August 2020, total 157 GC patients (69 RDG, 88 LDG) at Kurume University Hospital were identified. Table 1 summarizes the clinicopathological characteristics of all patients and matched patients. Before PSM, the mean age of the RDG group was determined to be significantly younger than that of the LDG group (66.9 ± 10.2 and 72.3 ± 10.1 years, respectively; *P* < 0.001), and the mean BMI of the RDG group was significantly lower than that of the LDG group (22.4 ± 3.5 and 23.5 ± 3.1 kg/m^2^, respectively; *P* = 0.044). In addition, the ASA-PS score of the RDG group was significantly lower than that of the LDG group (*P* = 0.005). There were no significant differences between groups in terms of gender, previous abdominal operation, comorbidity, or tumor status. Based on the PSM, we selected 50 patients who underwent RDG and 50 patients who underwent LDG. After adjusting for background factors by PSM, the patient distributions were well balanced between the RDG and LDG groups. In both groups, stage I cancer rate was 68 %.

**Table 1:** Clinicopathological findings before and after matching

	Entire cohort		*P* value	Propensity-score matched cohort		*P* value
	RDG (n = 69)	LDG (n = 88)		RDG (n = 50)	LDG (n = 50)	
Age (years)	66.9 ± 10.2	72.3 ± 10.1	0.001*	69.2 ± 1.4	69.3 ± 1.4	0.942
Gender						
Male	45 (65.2%)	58 (65.9%)	0.928	31 (62.0%)	34 (68.0%)	0.529
Female	24 (34.8%)	30 (34.1%)		19 (38.0%)	16 (32.0%)	
BMI (kg/m^2^)	22.4 ± 3.5	23.5 ± 3.1	0.044*	23.0 ± 3.6	22.9 ± 2.7	0.893
Previous abdominal operation						
No	47 (68.1%)	57 (64.8%)	0.660	32 (64.0%)	33 (66.0%)	0.834
Yes	22 (31.9%)	31 (35.2%)		18 (36.0%)	17 (34.0%)	
Comorbidity						
No	22 (31.9%)	17 (19.3%)	0.071	13 (26.0%)	14 (28.0%)	0.822
Yes	47 (68.1%)	71 (80.7%)		37 (74.0%)	36 (72.0%)	
ASA PS score						
I	18 (26.1%)	16 (18.2%)	0.005*	12 (24.0%)	13 (26.0%)	0.886
II	48 (69.6%)	52 (59.1%)		35 (70.0%)	33 (66.0%)	
III	3 (4.3%)	20 (22.7%)		3 (6.0%)	4 (8.0%)	
Tumor size (mm)	30.0 ± 14.6	35.0 ± 23.3	0.120	31.8 ± 15.1	30.4 ± 17.6	0.666
Histological type						
Differentiated	38 (55.1%)	47 (53.4%)	0.834	28 (56.0%)	26 (52.0%)	0.688
Undifferentiated	31 (44.9%)	41 (46.6%)		22 (44.0%)	24 (48.0%)	
pT classification						
T1	48 (69.6%)	55 (62.5%)	0.423	33 (66.0%)	33 (66.0%)	0.853
T2	8 (11.6%)	7 (8.0%)		5 (10.0%)	3 (6.0%)	
T3	6 (8.7%)	10 (11.4%)		6 (12.0%)	8 (16.0%)	
T4	7 (10.1%)	16 (18.2%)		6 (12.0%)	6 (12.0%)	
pN classification						
N0	56 (81.2%)	59 (67.1%)	0.071	38 (76.0%)	39 (78.0%)	0.976
N1	3 (4.4%)	11 (12.5%)		3 (6.0%)	3 (6.0%)	
N2	7 (10.1%)	7 (8.0%)		6 (12.0%)	6 (12.0%)	
N3	3 (4.4%)	11 (12.5%)		3 (6.0%)	2 (4.0%)	
pStage						
I	51 (73.9%)	56 (63.6%)	0.252	34 (68.0%)	34 (68.0%)	0.937
II	11 (15.9%)	15 (17.1%)		9 (18.0%)	10 (20.0%)	
III	7 (10.1%)	17 (19.3%)		7 (14.0%)	6 (12.0%)	

*BMI* body mass index, *ASA* American Society of Anesthesiologists, *PS* performance status

*P < 0.05 indicates statistical significance

### Surgical outcome

Table 2 summarizes the details of the surgical outcomes in the matched groups. No significant between-group differences were determined in the degree of LN dissection or method of reconstruction. None of the operations were converted to an open procedure in either group, or there was no laparoscopic conversion in the RDG group. The operation time for the RDG group (350.1 ± 58.1 min) was significantly longer than that for the LDG group (257.5 ± 63.7 min; *P* < 0.0001). The difference between both groups in the mean number of retrieved LNs, blood loss, maximum plasma CRP levels, and D.AMY concentration in drainage fluid on postoperative day 3 was not significant. The median postoperative hospital stay was slightly shorter in the RDG group than that in the LDG group (12.0 ± 5.6 vs. 13.0 ± 12.3 days; *P* = 0.038). No treatment-related deaths and rehospitalizations were recorded.

**Table 2:** Details of surgical outcomes in the matched groups

	RDG (n = 50)	LDG (n = 50)	*P* value
Lymph node dissection			
D1	2 (4.0 %)	3 (6.0 %)	0.790
D1+	26 (52.0 %)	23 (46.0 %)	
D2	22 (44.0 %)	24 (48.0 %)	
Number of retrieved LNs	45.7 ± 18.1	44.9 ± 16.8	0.819
Reconstruction method			
BI	35 (70.0 %)	25 (50.0 %)	0.107
BII	3 (6.0 %)	7 (14.0 %)	
RY	12 (24.0 %)	18 (36.0 %)	
Open conversion	None	None	
Operation time (min)	350.1 ± 58.1	270.5 ± 63.7	< 0.0001*
Blood loss (ml)	12.5 ± 70.1	15.0 ± 36.3	0.234
CRP (mg/dl)	12.0 ± 5.5	11.7 ± 6.5	0.606
D.AMY (U/L)	96.0 ± 318.6	144.0 ± 498.2	0.105
Postoperative hospital stay (days)	12.0 ± 5.6	13.0 ± 12.3	0.038*
Mortality	None	None	

*BI* Billroth I reconstruction, *BII* Billroth II reconstruction, *RY* Roux en Y reconstruction, *LN* lymph node, *CRP* C-reactive protein, *D.AMY* drain amylase

*P < 0.05 indicates statistical significance

### Postoperative complications

Table 3 details the incidence of postoperative complications. In total, 7 patients (14.0 %) incurred complications after RDG, compared with 10 patients (20.0 %) who experienced complications after LDG (*P* = 0.425). The incidence of postoperative complications was nearly similar in both RDG and LDG groups. There tended be fewer pancreatic fistula and severe complications (CDC grade III or higher) in the RDG group as compared with the LDG group (2.0 and 10.0 %; *P* = 0.092; 2.0 and 14.0 %; *P* = 0.061, respectively). Only one (2.1 %) patient in the RDG group had a CDC grade IIIa surgical complication (anastomotic leakage). No patient in either group required reoperation or surgical intervention.

**Table 3:** Postoperative complications in the matched groups

	RDG (n = 50)	LDG (n = 50)	*P* value
Postoperative complications			
Anastomotic leakage	2 (4.0 %)	1 (2 %)	0.558
Pancreas fistula	1 (2.0 %)	5 (10.0 %)	0.092
Leakage of lymphatics	2 (4.0 %)	0	0.153
Intra-abdominal abscess	1 (2.0 %)	2 (4.0 %)	0.558
Pneumonia	0	1 (2.0 %)	0.315
Other	4 (8.0 %)	3 (6.0 %)	0.695
Cholecystitis	0	1	
Liver infarction	1	0	
Liver abscess	0	1	
Stomal ulcer	2	0	
Delayed gastric emptying	1	1	
Complication grade (CDC)			
Grade II	6 (12.0 %)	3 (6.0 %)	0.061
Grade III	1 (2.0 %)	7 (14.0 %)	
Grade IV/V	0	0	

*CDC* Clavien–Dindo classification

*P < 0.05 indicates statistical significance

### Total cost

In the RDG group, the total medical cost, including the operative fee and admission expense, was 1,808,998 (1,595,166–2,465,620) JPY, and in the LDG group, it was 1,836,041 (1,509,508–4,103,092) JPY (*P* = 0.195).

## Discussion

In recent years, minimally invasive surgery has been rapidly developed and improved, thereby providing a new alternative surgical method for the treatment of early-stage GC, for which it has gradually become the mainstream surgical treatment [2, 3]. However, there are still some technical limitations to laparoscopic surgery, so the minimally invasive approach for advanced GC that requires D2 LN dissection has not achieved a worldwide consensus yet [15, 16]. Robotic surgery has advantages over conventional laparoscopic surgery in terms of tremor filtering, clarity of the surgical view in three dimensions, and improved dexterity with the articulated arm [8, 15]. RG is an alternative minimally invasive surgical technique that has been gradually used for the treatment of GC [17, 18]. However, clinically significant benefits of RG have yet to be sufficiently proven.

Several studies have compared RDG and LDG, but the outcomes of some of these studies differed in terms of operative time, blood loss, number of resected LNs, and rate of complications [2, 10, 11, 19, 20]. This study showed that the operative time was significantly longer in the RDG group as compared with the LDG group, which may be attributed to the additional time required for docking and preparation and reduced surgeons’ less experience with the use of this novel robotic modality. Operation time was also longer when LDG was initially adopted. As surgeons have gained experience using this technique, the operation time for LDG has gradually decreased. Similar trends may be anticipated for RDG. Some studies have found no difference in operation time between robotic and laparoscopic surgery when performed by an experienced surgeon. Huang et al. reported that both operating time and docking time decreased and stabilized after 25 procedures for RG and after 41 procedures for laparoscopic gastrectomy for experienced surgeons [21]. The learning curve for the da Vinci surgical system is extremely short for surgeons, with sufficient experience performing conventional laparoscopic gastrectomy.

Our study showed that the difference in the mean number of retrieved LNs and the mean amount of blood loss between groups was not significant. Several studies have reported the equivalence of RDG and LDG in terms of blood loss and LN dissection [11, 15–20]. The articulating instrument might have been responsible for the higher number of resected LNs, because it aids surgeons in reaching deep areas that would be otherwise unreachable when using laparoscopic straight forceps. In particular, articulating instruments could prevent injuries to the blood vessels, such as the common hepatic artery, celiac trunk, and splenic artery, and allow precise dissection, particularly in the inferior pylorus area and the suprapancreatic area, which are technically demanding in laparoscopic surgery. In this study, a prolonged operative time did not affect the short-term surgical outcomes. In other words, although RDG requires a longer operative time as compared with LDG, the need for less surgical manipulation during robotic surgery may lead to lower surgical stress among patients.

When assessing the quality and safety of operations, determining any presence of postoperative complication is also an important consideration [22, 23]. Obama et al. reported no differences in the incidence of overall or major complications between patients undergoing RDG and LDG [24]. On the other hand, in their multi-institutional prospective study, Uyama et al. reported the advantages of RDG in terms of reduced postoperative complications [9]. In this study, we observed that the incidence of postoperative complications was almost similar between the RDG and LDG groups. However, there tended to be a lower rate of pancreatic fistula in the RDG group as compared with the LDG group. Pancreatic fistula, which is one of the most troublesome and potentially lethal complications after gastrectomy, results from injury to the pancreatic tissue during the surgical manipulation of the inferior pylorus area and the suprapancreatic area with excessive retraction of the pancreatic body. The reason for the lower rate of this complication in our study might be that the meticulous precision of the robotic articulated arm has likely reduced injury to the pancreas. However, major complications (CDC grade ≥ IIIa) observed in seven cases (14.0 %) were determined to be more frequent in the LDG group than those observed in only one case (2.3 %) in the RDG group. In their study, Hyun et al. reported that the RDG group had a higher total number of complications compared with the LDG group, but most of those complications were minor and could be treated nonsurgically [25]. As a result of fewer severe complications, the duration of the postoperative hospital stay was shorter in the RDG group as compared with the LDG group.

The perceived additional cost associated with robotic surgeries is a common issue. The total difference in cost between the LDG and RDG groups has been predicted to be around 3,900 USD, which is mainly derived from the robotic system itself [26]. Although robotic surgery device is currently monopolized by one company, hopefully in the near future, the cost will reduce when similar devices are developed and sold by other companies. In this study, the total medical cost was almost similar between the RDG and LDG groups. Another prospective study by Uyama et al. has shown that complications are associated with increased cost [9]. Reduced length of ICU admission or overall stay has an effect on the cost [27]. Not only do postoperative complications lead to a longer hospital stay and higher medical expenses but also, they have been reported to worsen the survival rate after curative gastrectomy [21, 28]. Most previous reports have failed to demonstrate clear clinical advantages of RDG over LDG [11, 18–20]. However, in this study, we identified the benefits of RDG for reducing morbidity. A reduced morbidity by robotic surgery may decrease the total medical cost and duration of hospital stay and improve the quality of life and survival rate of patients.

This study had several limitations. First, it was a single-institute, retrospective study. Although a randomized controlled trial was not performed, we used PSM analysis to reduce selection bias as far as possible, but the patient backgrounds cannot be fully corrected. Second, the study’s sample size was not large enough, and more than half of the patients had stage I cancer. Finally, we did not investigate long-term oncological outcomes, including late complications, recurrence, or survival.

## Conclusions

This study demonstrated that RDG is a technically feasible and safe procedure for the treatment of patients with early-stage GC in terms of short-term surgical outcomes. We believe that, in the near future, RDG may be a practical and feasible alternative to LDG for the treatment of GC. However, whether RDG is truly superior to LDG requires verification via large-scale randomized controlled trials.

## Data Availability

All data are included in the manuscript. The datasets used and analyzed during the current study available from the corresponding author upon reasonable request.

## Abbreviations

ASA-PS: American Society of Anesthesiologists performance status
BMI: Body mass index
CDC: Clavien–Dindo classification
CRP: C-reactive protein
D.AMY: Drain amylase
GC: Gastric cancer
JCGC: Japanese classification of gastric carcinoma
LDG: Laparoscopic distal gastrectomy
LNs: Lymph nodes
RDG: Robotic distal gastrectomy
PSM: Propensity score-matching
